# Prognostic Significance of NT-proBNP Levels in Patients over 65 Presenting Acute Myocardial Infarction Treated Invasively or Conservatively

**DOI:** 10.1155/2015/782026

**Published:** 2015-10-11

**Authors:** Wojciech Drewniak, Wojciech Szybka, Dariusz Bielecki, Michal Malinowski, Joanna Kotlarska, Agnieszka Krol-Jaskulska, Agata Popielarz-Grygalewicz, Aleksandra Konwicka, Marek Dąbrowski

**Affiliations:** Cardiology Clinic of Physiotherapy Division of The 2nd Faculty of Medicine, Medical University of Warsaw, Bielanski Hospital, Ceglowska 80 Street, 02-903 Warsaw, Poland

## Abstract

*Objectives*. Assessment of prognostic significance of NT-proBNP level and the effects of invasive (I) and conservative (C) treatment of acute myocardial infarction (AMI) in patients over 65. *Materials and Methods*. One-year survival was assessed in 286 consecutive patients with AMI aged 65–100 (79 ± 8) subjected to I or C treatment (136 and 150 individuals), respectively. *Results*. 245 (85%) patients survived in-hospital stay: 124 (91.1%) received I treatment and 121 (80.6%) received C treatment. Heart failure (HF) was diagnosed in 30 patients receiving I treatment (22.6%) and in 71 subjected to C treatment (47.3%), *p* < 0,0001. NT-proBNP levels in the latter group were significantly higher than in the 185 patients without HF (12311 ± 13560 pg/mL versus 4773 ± 8807 pg/mL, *p* < 0.0001). NT-proBNP levels after coronary angioplasty were lower than in patients receiving C treatment (5922 ± 10250 pg/mL versus 8718 ± 12024 pg/mL, *p* = 0.0002). Left ventricular ejection fraction was significantly higher in I patients than in C patients (47 ± 13% versus 42 ± 11.6%, *p* = 0.004). During the one-year follow-up, 82.3% of I patients and 61.2% of the C patients survived (*p* < 0.0003). There was a significantly lower probability of death at NT-proBNP below 8548.5 pg/mL. *Conclusions*. The NT-proBNP level in the first day of AMI is a good prognosticator. One-year follow-up prognosis for patients who received I treatment in the AMI is better than that for C patients. I patients exhibit superior left ventricular function after angioplasty and in the follow-up.

## 1. Introduction

Postinfarction heart failure is an increasing medical and epidemiological problem, especially in the group of patients aged over 65 years, despite the ever more effective treatments available in the acute phase of myocardial infarction [1]. Elderly persons are afflicted with impaired relaxation of the ventricular cardiac muscle leading to elevated end-diastolic pressure and increased NT-proBNP secretion, symptoms affected also by deteriorating kidney function and the associated hypervolemia. Given the large number of factors contributing to increased natriuretic peptide levels in the elderly, the normal NT-proBNP ranges in persons over 65 greatly exceed those in the younger age groups [2, 3]. While NT-proBNP levels have been confirmed as a good prognostic indicator in younger age groups, the same still needs to be verified in the 65+ age group. The objective of this study was to assess the possibility of predicting an adverse course of the disease in its early stages based on results of commonly performed tests, such as NT-proBNP levels and left ventricular ejection fraction (EF%) in invasively or conservatively treated patients over 65. We also have assessed the effects of invasive versus conservative therapies applied in the acute phase of myocardial infarction in patients over 65 on the patients' survival and on the incidence of cardiac insufficiency over a one-year follow-up period.

## 2. Materials and Methods

The study involved 286 consecutive patients aged 65–100 (79 ± 8 on average). The patients, suffering from myocardial infarction confirmed by elevated troponin I levels, were admitted to the cardiologic intensive care unit. 139 of their number were diagnosed with ST-elevation myocardial infarction in ecg (STEMI), while the remaining 147 were diagnosed with non-ST elevation myocardial infarction (NSTEMI). 136 patients were subjected to invasive therapy (PCI), while 150 received conservative treatment. STEMI patients were fitted for invasive strategy within 12 hours from first medical contact. All of them had written consent for this way of therapy. In NSTEMI patients, “time window” was distended to 48 hours and related to risk stratifications criteria, as chest pain, ST in ecg, and troponin I dynamics, as well as coexistence of diabetes, heart failure, or mild renal insufficiency. Patients in cardiogenic shock, chronic heart failure in NYHA class IV before hospitalization, and renal failure with GFR ≤ 30 mL/min/1.73 m^2^ were excluded from analysis. Patients were prescribed conservative treatment when it was already too late to resort to invasive treatment strategy or if they did not consent to coronarography and PCI when these were required. Patients from this group were not subjected to fibrinolysis. 71 of the patients (24.83%) additionally suffered from diabetes, and 186 (65.03%) suffered from arterial hypertension. 57.7% of the patients had previously been diagnosed with coronary heart disease, while 26.6% had already in the past suffered from myocardial infarction. 52 of the patients (18.2%) displayed symptoms of serious heart failure (Killip II/III-IV) at admission to hospital (Table 1). Nearly all patients received optimal pharmacologic therapy: antiplatelet 100%, beta blocking 83,9%, ACE inhibitors 74,4%, and statins 91,2%; it was ordered according to their clinical status.

The patients were followed up for 12 months, and their survival, NT-proBNP levels, and physical efficiency were determined at six-month intervals. Primary: end-point was all cause mortality in one year follow-up. Secondary: NT-proBNP level and left ventricular ejection fraction were determined in follow-up period.

NT-proBNP levels in blood serum were determined using the Roche test. Given the very large number of left venricular parametres we have chosen left venticular ejection fraction as simple and generaly acceptable. The ejection fraction (EF) was assessed by echocardiography based on Simpson's method.

### 2.1. Statistical Methods

The parametric Student's* t*-test and the nonparametric Wilcoxon and chi-square tests were used to study differences between the respective patient groups. Regression analysis and Cox model multifactor analysis were applied to study relationships between variables. The patients' survival was illustrated with Kaplan-Maier curves for the various NT-proBNP level quartiles. Analyses were performed using the JMP statistical program from SAS Institute.

## 3. Results

**Figure 1:**
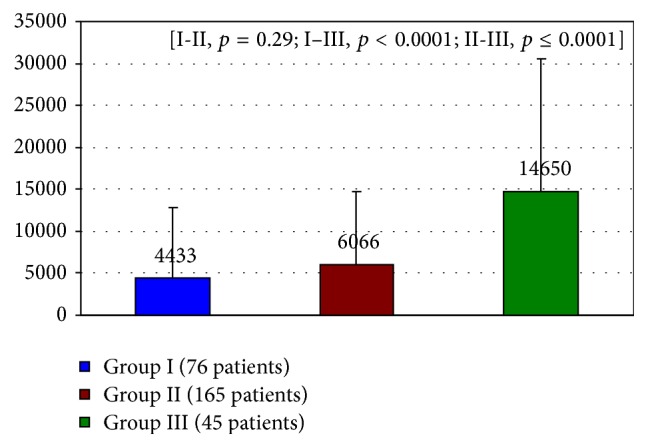
Mean NT-proBNP level estimated in acute phase in relation to age groups. Group I: 65–75 years. Group II: 76–85 years. Group III: over 85 years.

Patients receiving invasive treatment were hospitalized for 3–5 days, while those treated conservatively were hospitalized for 10–14 days. 30-day survival was 86% (245 patients), including 124 (91.1%) of those treated invasively and 121 (80.6%) of those treated conservatively. Reason of the death of 41 was heart failure and all of them were in hospital. The mean NT-proBNP level for the entire group of studied patients determined within the 48-hour period following the onset of myocardial infarction pain was 7389 ± 13892 pg/mL and was strongly related to patients' age and was tending to increase with the patients' age (Figure 1). The observed differences were statistically highly significant. The left ventricular ejection fraction (EF) assessed in the 2nd to 5th day of myocardial infarction was 44.4 ± 14.7% on average.

**Figure 2:**
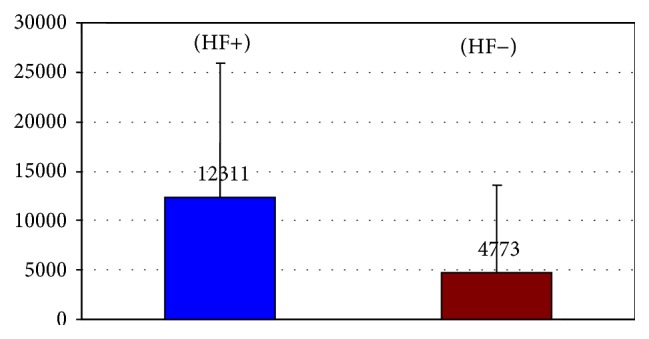
Comparison of NT-proBNP levels estimated after admission in patients with (HF+) and without heart failure (HF−).

The NT-proBNP levels in 101 patients diagnosed with HF (Killip II/III-IV), including patients with heart failure symptoms at admission to hospital and 49 patients who developed HF during their hospital stay, were significantly higher than those in the 185 patients who did not suffer from heart failure (12311 ± 13560 pg/mL versus 4773 ± 8807 pg/mL, *p* < 0.0001) (Figure 2). This group comprised 30 patients (22.6%) treated invasively and 71 patients (47.3%) who received conservative treatment (OR for HF in invasively treated patients was 0.23 (95% CI: 0.19–0.51); *p* = 0.05). Compared to the situation at admission to hospital, additional 9 patients treated invasively and 40 patients treated conservatively developed symptoms of heart failure during hospitalization. The initial NT-proBNP levels in invasively treated patients were lower than in the patients receiving conservative treatment who were tested in the first day of hospitalization within 48 hours from the onset of myocardial infarction pain (5922 ± 10250 pg/mL versus 8718 ± 12024 pg/mL, *p* < 0.0002).

The mean left ventricular ejection fraction figures determined within 2–5 days from myocardial infarction were significantly higher in invasively than in conservatively treated patients (47 ± 13% versus 42 ± 11.6%,  *p* = 0.004) (Table 2).

Bearing in mind that single-variant analysis showed NT-proBNP levels to be dependent on patients' age and since the invasively treated patients were younger, multivariant analysis was performed to determine the effect of ejection fraction, age, and clinical symptoms of heart failure on NT-proBNP levels. Each of these factors was found to have exerted an independent and significant effect on NT-proBNP levels (age, *p* = 0.00009; ejection fraction, *p* = 0.0016; and clinical symptoms of heart failure, *p* < 0.0001).

### 3.1. Six-Month Follow-Up Period

112 (82.3%) of the invasively treated patients and 100 (66.44%) of patients who received conservative treatment were alive in six-month follow-up period (*p* = 0.008). The NT-proBNP levels continued to be significantly lower in the former group of patients (919 ± 1804 pg/mL versus 2336 ± 3464 pg/mL, *p* = 0.0003), while the ejection fraction figures were still higher (51.4 ± 8.3% versus 48.3 ± 10.4%, *p* = 0.03) (Table 3).

### 3.2. Twelve-Month Follow-Up Period

84 (29.3%) of the followed up patients died during the 12-month period, leaving 82.3% of the invasively treated and 61.2% of the conservatively treated patients alive (*p* < 0.0003). These figures confirm a 26.9% reduction of one-year mortality in the group of patients subjected to invasive treatment. All the deaths in the invasively treated group were recorded during the first six months of follow-up. The study evaluated total mortality. The NT-proBNP levels remained significantly lower in the invasively treated patients compared to patients receiving conservative treatment (922 ± 1782 pg/mL versus 2107 ± 4248 pg/mL, *p* = 0.002). Further drops in NT-proBNP were observed only in the conservatively treated patients, however. The left ventricular ejection fraction continued to remain higher in the invasively treated patients than in the conservative treatment group (52.6 ± 8.3% versus 48.9 ± 9%, *p* = 0.01) (Table 4).

### 3.3. Death Risk Factors in the 12-Month Follow-Up Period

**Figure 3:**
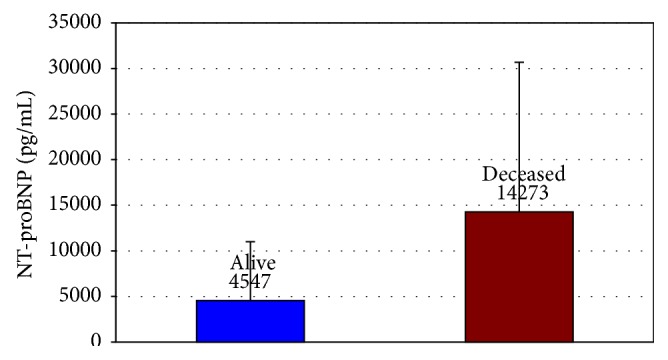
Comparison of NT-proBNP levels estimated at admission in patients deceased and alive after 12 months.

The NT-proBNP levels determined in the acute phase of the disease were significantly higher in the group of deceased patients after 12 months than in the group of those who were alive a year after their myocardial infarction (14273 ± 16419 pg/mL versus 4547 ± 6468 pg/mL, *p* < 0.0001) (Figure 3). The average age of the deceased patients was 84 ± 9 years and was significantly higher than the age of patients who were alive 12 months after the acute coronary syndrome (78 ± 7 years, *p* < 0.0001).

**Figure 4:**
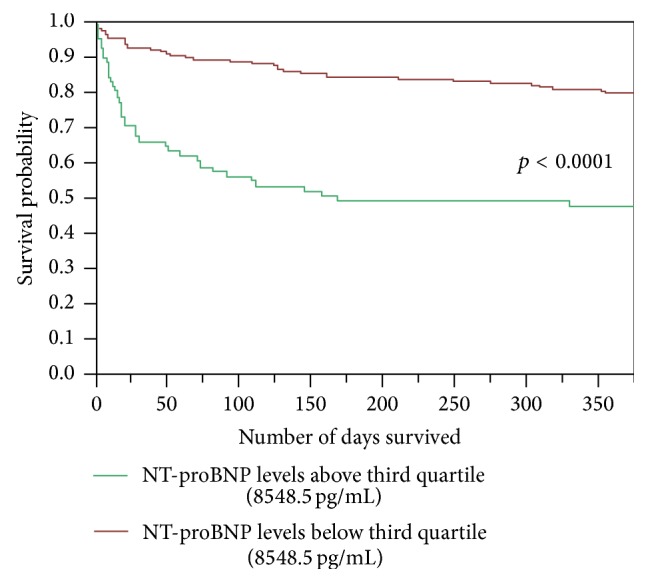
Kaplan-Meier curves illustrating survival for the various NT-proBNP level quartiles.

Probability of death was determined by baseline NT-proBNP denoted to 48 hours of the onset of chest pain. The Kaplan-Meier curves illustrating survival probability for the various NT-proBNP level quartiles show the probability of death to be significantly lower when these figures drop below 8548.5 pg/mL (Figure 4).

Multivariable analysis involving NT-proBNP levels in blood serum in the acute phase of the disease, the left ventricular ejection fraction determined 2 to 5 days after admission to hospital, and the age of patients was performed to identify risk factors discernible in the early stage of myocardial infarction which adversely affect the subsequent course of the disease. The results show that NT-proBNP levels, the patients' age, and the left ventricular ejection fraction are all independent death risk factors (Table 5).

### 3.4. Study Limitations

The invasively treated patients were younger than those treated conservatively.

## 4. Discussion

Elderly patients face higher risks when affected by acute coronary syndrome. The reasons for this may include the greater extent of atherosclerosis in coronary arteries and peripheral vessels as well as numerous other diseases the patients may be suffering from at the time. Due to the nontypical nature of symptoms, and probably because of “psychological barriers,” elderly patients suffering from acute coronary syndrome often delay first medical contact, thus preventing the use of the most effective therapies. This mechanism was confirmed in the group of patients we studied: upwards of half of their number (51%) were hospitalized more than 12 hours after the onset of symptoms. In most cases, this delay resulted from prolonged hospital preadmission procedures, often also because the elderly patients had problems in precisely identifying the onset of the coronary incident; moreover, some of them refused to consent to invasive treatment. Chronic and frequently recurring conditions such as dyspnoea, asthenia, or poorly describable chest pains may be additional circumstances disguising acute coronary syndrome. Although persons suffering from this syndrome account for increasing percentages of patients treated in cardiologic intensive care units, limited amounts of data are available concerning optimal treatments of these patients aged 65 and over. Improved prognosis in early hospitalization means that increasing numbers of patients survive this stage. Higher numbers of gravely ill patients surviving the acute phase of myocardial infarction, albeit often with severe damage to the left ventricle suffered despite the medical intervention, mean there are increasing numbers of patients who may potentially develop symptoms of heart failure, mainly due to impaired left ventricular contractility. Heart failure in the elderly leads to increased mortality, recurring hospitalization, and deterioration of life comfort [1]. Primary angioplasty is seen to be the best method of preventing postinfarction cardiac insufficiency. Although age was determined to be an independent risk factor decreasing the likelihood of positive outcomes in the treatment of acute coronary syndrome, there appear to be virtually no treatment recommendations specifically in respect of this particular group of patients that would be accepted in medical practice [4]. The reason for this is that elderly patients are poorly represented in large-scale clinical and observational studies on which treatment recommendations are based [5]. On the other hand, although the benefits of invasive strategies in myocardial infarction treatment are well documented, this fact is not always acted upon in the case of patients aged over 65 [6, 7]. The inferior overall health picture in elderly patients and the more severe clinical course of myocardial infarction, due to chronic illnesses such as diabetes, arterial hypertension, chronic coronary heart disease (often affecting multiple arteries), heart failure, and also kidney insufficiency, prompt many doctors to postpone or entirely abandon invasive treatment. Meanwhile, retrospective analyses demonstrate that elderly patients benefit from invasive treatment to a significant extent [8–10] which suggests that decisions to deny such treatment to patients suffering from myocardial infarction because of their other diseases are taken too lightly. We assessed the influence of invasive and traditional therapy on survival and postinfarction heart failure in elderly group. NT-proBNP levels determination is by now a universally acknowledged biochemical method of testing cardiac insufficiency. High NT-proBNP level is a significant death risk factor in younger patients suffering from ischemic heart disease [11, 12]. The prognostic significance of increased NT-proBNP levels in the course of acute coronary syndrome in persons aged below 65 years is especially well documented. On the other hand, data on the prognostic significance of NT-proBNP level in patients over 65 suffering from acute coronary syndrome are scarce in the available literature. It was demonstrated, however, that high levels of NT-proBNP in elderly patients hospitalized with their first ever cardiac insufficiency episode are indicative of increased cardiovascular risks in the follow-up period [12]. Also indicated was the increased risk of no-reflow phenomenon following angioplasty. In our group of invasively treated patients, the NT-proBNP levels in the acute phase of myocardial infarction (but already just after angioplasty) were significantly lower than those in the group of conservatively treated patients, and this difference continued to persist after 6 and 12 months of observation. It must be stressed here that although the mean NT-proBNP levels decreased significantly, they nevertheless remained excessively elevated, exceeding severalfold the accepted normal level. The NT-proBNP levels in the patients we analyzed were at least several or even more than ten times higher than the levels reported in the already quoted publications. One must bear in mind, however, that we selected our group of patients based on age and that in some of them myocardial infarction resulted in additional complications in the form of symptomatic acute heart failure which significantly affected the NT-proBNP levels determined in the acute phase of the disease. The greater incidence of heart failure in patients of the age group we studied is also worth recalling. Like other authors before us, we demonstrated a significant correlation between NT-proBNP levels on one hand and age and decreased left ventricular ejection fraction on the other hand. In our group comprising patients receiving either conservative or invasive treatment we found NT-proBNP level to be an independent death risk factor in the 12-month follow-up period.

Echocardiography is a simple and commonly available tool serving to assess the left ventricular function. In the initial stage of myocardial infarction systolic function was significantly superior in the invasively treated group compared to the group receiving conservative treatment, and this difference in ejection fraction still persisted 6 and 12 months later. The restoration of patency in the coronary artery responsible for the developing myocardial infarction probably has a direct effect on the left ventricular ejection fraction as it improves contractility in the ischemic zone threatened with necrosis. Adrenergic stimulation triggers a hyperkinetic contraction of the other left ventricle segments. Thereafter the ventricle probably undergoes a postinfarction remodeling that is more pronounced in conservatively treated patients, something that may be due to the greater extent of damage and may negatively impact the ejection fraction while also being conducive to an aggravation of cardiac insufficiency. This scenario is consistent with the “open artery” theory prevailing at the turn of the 1980s and 1990s and confirmed by numerous studies. The higher initial NT-proBNP levels also resulted in the persistence of left ventricular systolic dysfunction, as reflected by EF values, 6 and 12 months after the infarction. The important factors in prognostication of elderly patients include quality of life, self-reliance, and physical efficiency [13, 14]. The high NT-proBNP levels indicative of increased risk of death or disability may suggest that the more aggressive treatments ought to be considered as the first option to be followed by rehabilitation and pharmacotherapy adapted to the age of this group of patients as well as regular check-ups. Infrequent publications and congress papers have been appearing over the past several years reporting the benefits of preplanned doctor and nurse care provided on a regular basis to circulatory insufficiency patients at their homes using telemedical solutions. These benefits include increased sense of security in the patients and, thanks to ongoing monitoring and the possibilities to forestall disease aggravations, fewer and shorter hospitalizations and, most importantly, reduced mortality [15]. The implementation of monitoring programs for patients facing a high risk of postinfarction heart failure appears to be advisable as it may reduce mortality, improve life comfort, reduce hospitalizations, and bring down the costs of medical treatment. The early determination of NT-proBNP levels may be particularly useful in this context [16].

## 5. Conclusions

1. Invasive treatment of acute myocardial infarction patients aged 65 and over, in line with recommendations, involving primary coronary intervention with stent implantation (PCI), results in improved 12-month prognosis compared to conservatively treated patients.
2. NT-proBNP level determined in the acute phase of myocardial infarction in patients over 65 years old, the patients' age, and left ventricular ejection fraction are significant prognostic indicators of survival probability and course of the disease in the medium-term observation period.
3. Patients over 65 years of age who received invasive treatment in the acute phase of myocardial infarction displayed better left ventricular function and lower NT-proBNP levels in the initial stages of the disease and also after 6 and 12 months of observation.
4. NT-proBNP level in the acute phase of myocardial infarction depends on the patient's age, left ventricular function, and the presence of complications such as heart failure.

## Figures and Tables

**Table 1:** Description of patients receiving invasive and conservative treatment.

Number of patients	All patients 286 pts	Invasively treated patients 136 pts	Noninvasively treated patients 150 pts	*p*
Diabetes	24.83%	17.6%	31.3%	0.01
Arterial hypertension	65.03%	57.3%	72%	0.025
Serious HF at admission to hospital	18.2%	15.4%	20.7%	0.25
Previously diagnosed CHD	57.7%	52.2%	63.3%	0.056
Previous MI	26.6%	24.3%	28.6%	0.7
Female/male	47.5%/52.5%	43%/57%	51%/49%	
Average age	79 ± 8	77 ± 7	82 ± 8	<0.0001

**Table 2:** Initial test results.

	Invasive treatment	Noninvasive treatment	
Mean NT-proBNP levels	5922 ± 10250 pg/mL	8718 ± 12024 pg/mL	*p* = 0.0002
Mean EF%	47% ± 13%	42% ± 11.6%	*p* = 0.0004

**Table 3:** Results after 6 months.

	Invasive treatment	Noninvasive treatment	
Mean NT-proBNP levels	919 ± 1804 pg/mL	2336 ± 3464 pg/mL	*p* = 0.0003
Mean EF%	51.4 ± 8.3%	48.3 ± 10.4%	*p* = 0.0313

**Table 4:** Results after 12 months.

	Invasive treatment	Noninvasive treatment	
Mean NT-proBNP levels	922 ± 1782 pg/mL	2107 ± 4348 pg/mL	*p* = 0.021
Mean EF%	52.6 ± 8.3%	48.9 ± 9%	*p* = 0.0134

**Table 5:** Multivariable survival analysis using Cox's regression model.

Variable	Hazard ratio	Confidence range (95%)	*p* value
NT-proBNP levels (2 groups: above and below the third quartile border, 8548.5 pg/mL)	0.33	[0.13; 0.75]	0.0132
Age (2 groups: above and below the third quartile border, 82 years old)	0.44	[0.2; 0.9]	0.0319
EF (2 groups: above and below the third quartile border, 50%)	4.06	[2.26; 7.36]	0.0001
